# Concurrent Autophagy Inhibition Overcomes the Resistance of Epidermal Growth Factor Receptor Tyrosine Kinase Inhibitors in Human Bladder Cancer Cells

**DOI:** 10.3390/ijms18020321

**Published:** 2017-02-04

**Authors:** Minyong Kang, Kyoung-Hwa Lee, Hye Sun Lee, Chang Wook Jeong, Cheol Kwak, Hyeon Hoe Kim, Ja Hyeon Ku

**Affiliations:** Department of Urology, Seoul National University Hospital, Seoul 110-744, Korea; dr.minyong.kang@gmail.com (M.K.); lee12042@snu.ac.kr (K.-H.L.); solareclipss@hanmail.net (H.S.L.); drboss@gmail.com (C.W.J.); mdrafael@snu.ac.kr (C.K.); hhkim@snu.ac.kr (H.H.K.)

**Keywords:** bladder cancer, epithelial growth factor receptor, tyrosine kinase inhibitor, autophagy, synergistic effect

## Abstract

Despite the potential therapeutic efficacy of epithelial growth factor receptor (EGFR) inhibitors in the treatment of advanced stage bladder cancer, there currently is no clear evidence to support this hypothesis. In this study, we investigate whether the concurrent treatment of autophagy-blocking agents with EGFR inhibitors exerts synergistic anti-cancer effects in T24 and J82 human bladder cancer cells. Lapatinib and gefitinib were used as EGFR inhibitors, and bafilomycin A1 (BFA1), chloroquine (CQ) and 3-methyladenine (3-MA) were used as the pharmacologic inhibitors of autophagy activities. To assess the proliferative and self-renewal capabilities, the Cell Counting Kit-8 (CCK-8) assay and a clonogenic assay were performed, respectively. To examine apoptotic cell death, flow cytometry using annexin-V/propidium iodide (PI) was used. To measure the autophagy activities, the expression levels of LC3I and II was determined by Western blot analysis. To validate the synergistic effects of autophagy inhibition with EGFR inhibitors, we specifically blocked key autophagy regulatory gene ATG12 by transfection of small interference RNA and examined the phenotypic changes. Of note, lapatinib and gefitinib triggered autophagy activities in T24 and J82 human bladder cancer cells, as indicated by upregulation of LC3II. More importantly, inhibiting autophagy activities with pharmacologic inhibitors (BFA1, CQ or 3-MA) remarkably reduced the cell viabilities and clonal proliferation of T24 and J82 cells, compared to those treated with either of the agents alone. We also obtained similar results of the enhanced anti-cancer effects of EGFR inhibitors by suppressing the expression of ATG12. Notably, the apoptotic assay showed that synergistic anti-cancer effects were induced via the increase of apoptotic cell death. In summary, concomitant inhibition of autophagy activities potentiated the anti-cancer effects of EGFR inhibitors in human bladder cancer cells, indicating a novel therapeutic strategy to treat advanced bladder cancer.

## 1. Introduction

Urothelial carcinoma of the urinary bladder is one of the most common malignancies in urinary tracts. The disease includes muscle invasion and is very aggressive, requiring a radical cystectomy with bilateral pelvic lymph node dissection [1]. Despite the radical treatments, patients with muscle invasive bladder cancer (MIBC) that finally progress into metastatic diseases should be treated by a systemic therapy, such as a cisplatin-based chemotherapy [2]. However, no therapeutic strategies currently achieve the clinically-significant efficacy to improve survival outcomes, and metastatic bladder cancer is almost uniformly fatal [3].

Based on recent comprehensive analyses of the genomic landscape of bladder cancer patients, several mutated genes have been identified as possible therapeutic targets, including epithelial growth factor receptor (EGFR) gene [4]. EGFR mutation has been identified as the most commonly-mutated gene in bladder cancer patients [5]. In addition, EGFR inhibitors have been widely used in other solid organ tumors, such as lung cancer, with a great therapeutic effect [5]. In contrast to lung cancer, EGFR inhibitors have not shown a significant efficacy in bladder cancer patients; therefore, it is urgent to develop a novel therapeutic strategy to improve the effectiveness of EGFR inhibitors in bladder cancer treatment [5].

Owing to the various environmental stimuli that EGFR receives in the regulation of intracellular homeostatic processes, such as autophagy, the relationship between EGFR and autophagy activity has been studied [6]. For example, Wei et al. [7] demonstrated that active EGFR directly bound and phosphorylated Beclin 1 protein, leading to autophagy suppression. In addition, treatment with EGFR tyrosine kinase inhibitor (TKI) disrupted Beclin 1 phosphorylation, and autophagy activities were restored [7]. Considering that autophagy can act as pro-survival machinery under a harsh condition, enhanced autophagy activities by EGFR inhibitor treatment may contribute to drug resistance in bladder cancer.

Here, we investigate to determine whether the concurrent autophagy inhibition can overcome the drug resistance of EGFR inhibitors in human bladder cancer cells in vitro. Moreover, we explore the molecular mechanisms involved in the synergistic anti-cancer effects of autophagy inhibition combined with EGFR inhibitors.

## 2. Results

### 2.1. Non-Significant Anti-Cancer Effects of Epithelial Growth Factor Receptor (EGFR) Inhibitors on Human Bladder Cancer Cells

**Figure 1:**
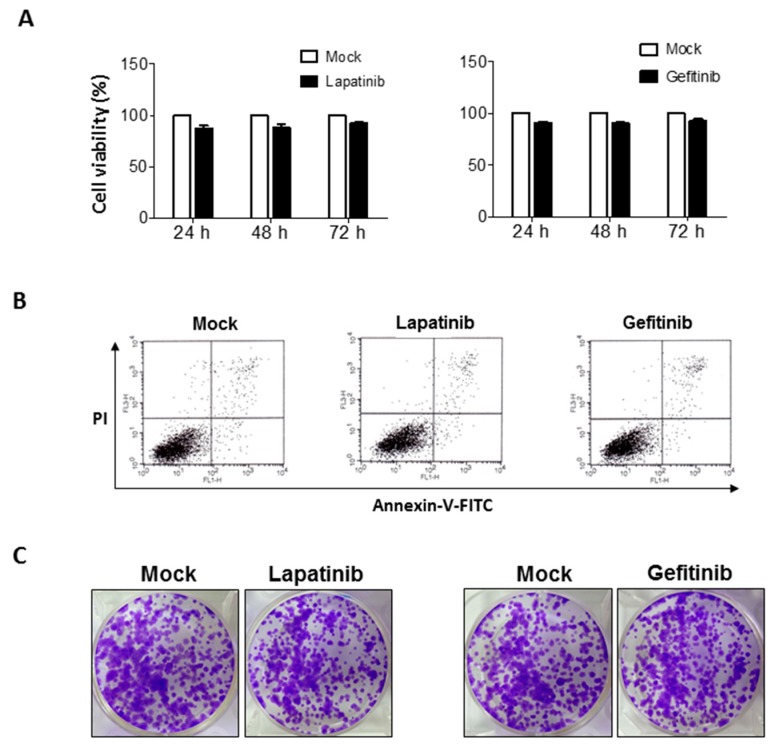
Limited effects of epithelial growth factor receptor (EGFR) inhibitors on T24 human bladder cancer cells. Evaluation of anti-cancer effects of lapatinib (5 μM) and gefitinib (5 μM) in T24 human bladder cancer cells by performing (**A**) a cell viability test at different treatment times (24, 48 and 72 h); and (**B**) flow cytometry analysis with annexin-V-FITC/PI double staining to determine apoptotic cell death after 24 h treatment of control or EGFR inhibitors; (**C**) limited efficacy of lapatinib (5 μM) and gefitinib (5 μM) in T24 human bladder cancer cells by performing the clonogenic assay. Colonies comprised of more than 50 individual cancer cells were visible and manually counted.

The EGFR inhibitors, lapatinib and gefitinib, were evaluated for anti-cancer effects on T24 human bladder cancer cells. When we treated T24 human bladder cancer cells with 5 μM of these EGFR inhibitors, the CCK-8 assay showed that neither lapatinib nor gefitinib could reduce cell viability in a time-dependent manner (Figure 1A). Apoptotic cell death did not significantly increase after treatment with either of the EGFR inhibitors (5 μM) for 24 h, compared to that in the negative control when assessed with flow cytometry (Figure 1B). Clonogenic assay also revealed that there was no significant reduction of the self-renewal capabilities of T24 cells when treated with EGFR inhibitors (5 μM) for 10 to 14 days, compared to that in the negative control (Figure 1C). In bladder cancer cell line J82, lapatinib and gefitinib did not affect the self-renewal potential when measured by the clonogenic assay (Figure S1A).

### 2.2. Autophagy Activation by EGFR Inhibitors in Human Bladder Cancer Cells

**Figure 2:**
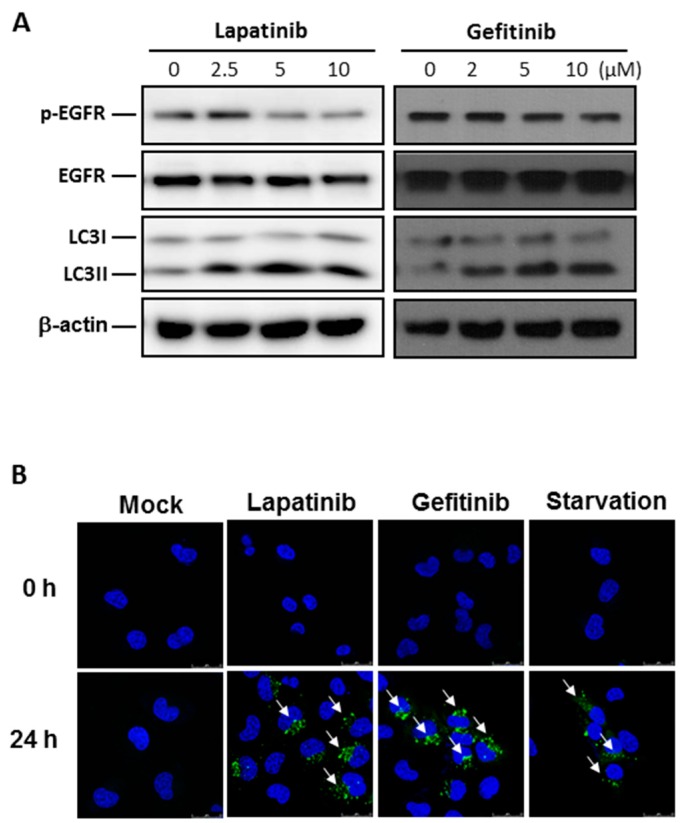
Epithelial growth factor receptor (EGFR) inhibitors significantly induce the autophagy activities in T24 human bladder cancer cells. (**A**) Western blot analysis for evaluating autophagy activation indicated by the expression of LC3I/II following treatment with EGFR inhibitors for 24 h, at different dosages (0, 2.5, 5 and 10 μM). β-actin was used as a loading control in the Western blot analysis; (**B**) confocal microscopic analysis of autophagy activation by the evaluation of green fluorescent protein (GFP) expression in LC3-GFP-expressing T24 cells after treatment of 5 μM EGFR inhibitors for 24 h. Cells cultured in serum-starvation conditions for 24 h were used as positive controls for autophagy activation analyses. White arrows indicate the LC3 autophagic puncta.

To examine autophagy activities, the expression levels of LC3 proteins were analyzed by Western blot analysis or immunostaining. Notably, it was found that treatments of lapatinib and gefitinib (0, 2.5, 5 and 10 μM) for 24 h triggered the autophagy activities in T24 human bladder cancer cells, as indicated by the upregulation of LC3II in Western blot analysis (Figure 2A). We further confirmed that EGFR treatment with the same experimental condition induced autophagy activation by observing increased GFP expression in T24 cells transfected with the LC3-GFP expression vector, as confirmed by confocal microscopy (Figure 2B). In J82 bladder cancer cells, LC3II expression also increased in a dose- and time-dependent manner after treatment with EGFR inhibitors (Figure S1B).

### 2.3. Synergistic Anti-Cancer Effects by Concurrent Treatment of EGFR Inhibitors and Autophagy Inhibitors in Human Bladder Cancer Cells

**Figure 3:**
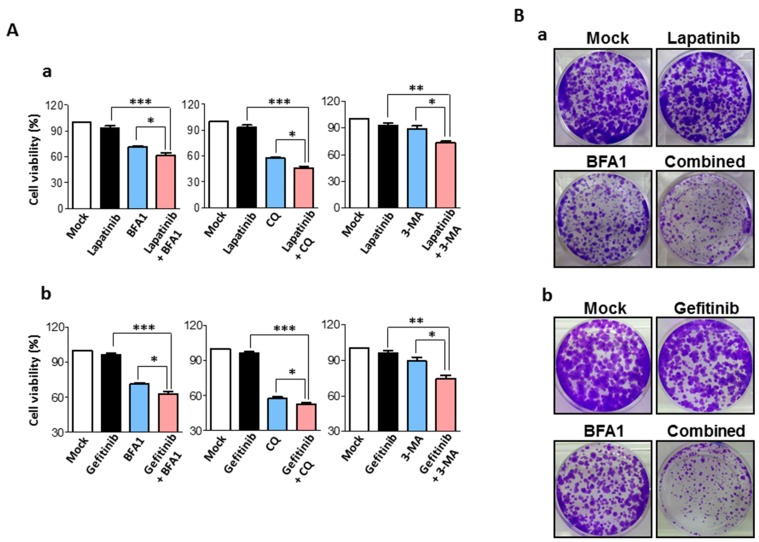
Synergistic anti-cancer effects of EGFR inhibitors and autophagy inhibitors on T24 human bladder cancer cells. (**A**) Evaluation of anti-cancer effects of (**a**) lapatinib and (**b**) gefitinib treatments in T24 human bladder cancer cells by cell viability tests under different treatment combinations (control, 5 μM of EGFR inhibitors (lapatinib; gefitinib), autophagy inhibitors and both agents) for 24 h. Autophagy inhibitors used in this study were 5 nM of bafilomycin A1 (BFA1), 10 μM of chloroquine (CQ) and 10 mM of 3-methyladenine (3-MA). Cell viability is shown as the mean percentage (%) of control ± SEM (*n* = 3, * *p* < 0.05, ** *p* < 0.01, *** *p* <0.001); (**B**) clonogenic assay determining the effects of concurrent treatments of (**a**) lapatinib (5 μM) and (**b**) gefitinib treatments (5 μM) with autophagy inhibitor BFA1 (5 nM) on clonal proliferation of T24 cells.

Considering that EGFR inhibitors activate autophagy in bladder cancer cells, we proceeded to determine whether concurrent inhibition of autophagy activities can enhance the anti-cancer effects of EGFR inhibitors. Notably, concurrent treatment of EGFR inhibitors (5 μM) and pharmacologic inhibition of autophagy by using bafilomycin A1 (BFA1) (5 nM), chloroquine (CQ) (10 μM) or 3-methyladenine (3-MA) (10 mM) for 24 h significantly reduced the cell viabilities of T24 and J82 cells, compared to those treated with either of the agents alone (Figure 3A and Figure S2Aa, respectively). We also observed that concurrent autophagy inhibition significantly enhanced the anti-clonal proliferation effects of EGFR inhibitors in T24 and J82 bladder cancer cells (Figure 3B and Figure S2Ab, respectively). These results indicate that the synergistic anti-cancer effects of these agents were induced by triggering apoptotic cell death.

### 2.4. Enhanced Anti-Cancer Effects of EGFR Inhibitors Combined with Genetic Inhibition of Autophagy Activities in Human Bladder Cancer Cells

**Figure 4:**
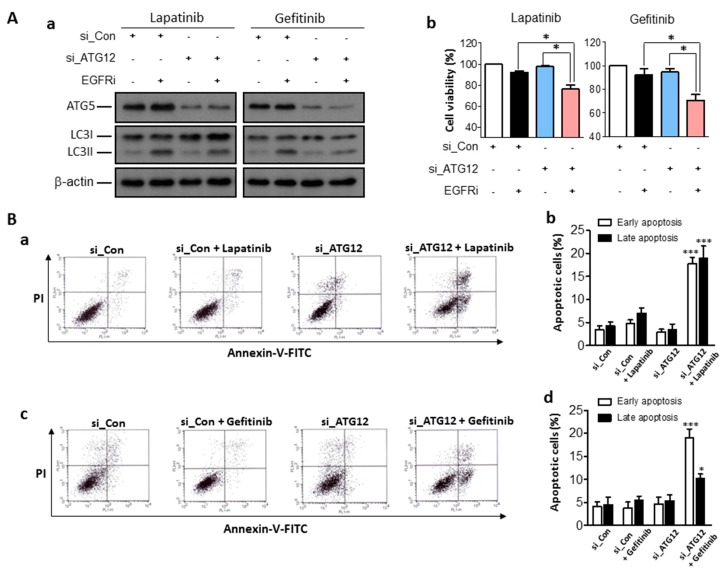
Effects of EGFR inhibitors with autophagy regulatory gene inhibition on T24 human bladder cancer cells. (**A**) (**a**) Western blot analysis to determine the knock-down efficiency and autophagy suppression by transfection of ATG12-siRNA (si_ATG12) in T24 cells, combined with the treatment of EGFR inhibitors (EGFRi). Scramble siRNA (si_Con; 50 nM) was used as a negative control of (si_ATG12). (**b**) Cell viability assay of T24 cells under the same treatment condition of EGFRi (5 μM) and si_ATG12 (50 nM) for 48 h; data are represented by the mean percentage of the control ± SEM (*n* = 3, * *p* < 0.05); (**B**) apoptosis assay by flow cytometry to examine the synergistic effects of EGFRi (5 μM) and genetic inhibition of autophagy activity by transfection of si_ATG12 (50 nM) for 48 h in T24 cells. (**a**,**b**) Lapatinib and (**c**,**d**) gefitinib were used as EGFRi. (**a**,**c**) representative results of the apoptosis assay and (**b**,**d**) quantitative data as bar graphs, indicating the proportion of early and late apoptotic cell death. Values are shown as the mean ± SEM (*n* = 3, * *p* < 0.05, *** *p* <0.001). si_Con (50 nM) was used as a negative control of si_ATG12.

We next determined whether the concurrent inhibition of autophagy activities by specific suppression of ATG12, one of the key transcription factors for LC3 expression, could also enhance the anti-cancer effects of EGFR inhibitors in human bladder cancer cells. ATG5, another autophagy regulatory gene, and LC3II expression were effectively inhibited by transfection of ATG12 siRNA (50 nM) for 48 h, despite the treatment with EGFR inhibitors in T24 cells (Figure 4Aa). Consequently, specific inhibition of ATG12 as an autophagy regulatory gene synergistically increased cell death when treated with 5 μM of lapatinib or gefitinib (Figure 4Ab). In J82 cells, genetic inhibition of autophagy activity synergistically reduced the cell viabilities when combined with 5 μM of gefitinib treatment (Figure S2B). Notably, we demonstrate that synergistic anti-cancer effects of the concurrent inhibition of autophagy activities with EGFR inhibitor treatments were triggered by increasing apoptotic cell death, as shown by flow cytometry analysis, indicating that synergistic effects were exerted by inducing apoptotic cell death (Figure 4B).

## 3. Discussion

To our knowledge, we are the first to report evidence that concurrent treatments of EGFR and autophagy inhibitors can significantly enhance the limited efficacy of EGFR inhibitors in human bladder cancer cells in vitro. Our study revealed that the synergistic growth inhibitory action between EGFR inhibitors and autophagy inhibitors was induced via apoptotic cell death in human bladder cancer cells.

EGFR is known as an oncogenic receptor tyrosine kinase (RTK), showing constitutive and abnormal upregulation in various types of malignancies [8]. EGFR plays a crucial role in cell proliferation, progression, invasion and angiogenesis [9]. Normal urothelial cells generally express EGFR, which is particularly associated with the dedifferentiation of cells, such as basal cells; thus, EGFR is frequently upregulated in urothelial cancer cells [10]. EGFR inhibitors have been suggested as novel therapeutic agents in advanced stages of cancer, refractory to conventional systemic chemotherapy [11]. Despite the potential benefits of EGFR inhibitors, some patients have primary resistance with limited efficacy of these drugs, while other patients experience secondary resistance to EGFR inhibitors [12]. Therefore, there have been unmet needs for the identification of novel strategies to overcome resistance to EGFR inhibitors in advanced cancer patients.

Autophagy is a critical intracellular pathway for regulating homeostasis, and it acts as pro-survival machinery under harsh environments [13]. Researchers have recently focused on dysregulated and unbalanced autophagy activities as one of the key features of cancers [14]. Interestingly, RTK (such as EGFR) activation regulates autophagy activity through multiple signaling pathways, and the autophagy induction induced by tyrosine kinase inhibitors (TKIs), such as EGFR inhibitors, has been considered as the drug-resistant mechanism of TKIs in advanced cancers [15]. Thus, recent studies have postulated a scientific rationale for the combination therapy of TKIs and autophagy inhibitors for overcoming acquired resistance of these drugs in several types of malignancies [16,17,18,19].

Consistent with our findings, Sugita et al. [18] revealed that gefitinib activated autophagy in non-small cell lung cancer cells (NSCLC; PC-9), and concurrent treatment with clarithromycin as an autophagy inhibitor significantly enhanced the anti-cancer effects of gefitinib. They also confirmed that knock-down of the *ATG5* gene as a key autophagy regulator efficiently yielded pronounced gefitinib-induced cytotoxicity in PC-9 cells [18]. In a study by Tang and colleagues, gefitinib-resistant cells (PC-9/gef), derived from long-term exposure in low-dose gefitinib, showed higher basal LC3-II levels compared to wild-type PC-9 cells (PC-9/wt) [19]. Notably, the combination of autophagy inhibitors, such as CQ and 3-MA, overcame the acquired resistance of gefitinib in PC-9/gef cells by activation of apoptotic cell death [19]. More importantly, the combined treatment of gefitinib and chloroquine was more effective than gefitinib alone in suppressing the tumor growth of PC-9/gef in the pre-clinical in vivo model [19]. In breast cancer cells, gefitinib treatment also induced autophagy activity along with the attenuation of the AKT and ERK1/2 signaling pathway, and complete inhibition of autophagy by *ATG7* siRNA transfection combined with gefitinib treatment created pronounced cytotoxicity compared to treatment with the respective single agents [17].

Conversely, a study by Li et al. showed that there was no activation of autophagy in TKI-resistant NSCLC cell lines under erlotinib treatment, and therefore, autophagy inhibition could not exert the synergistic effects with erlotinib in these cells [20]. Although the concurrent treatment with autophagy inhibitors and EGFR inhibitors has been attracting much attention as a novel anti-cancer strategy, it is still an open question whether autophagy inhibits cancer cell proliferation or promotes cancer cell survival under harsh circumstances, such as under TKI treatments. Several potential hypotheses of contradicting opinions are as follows: First, the molecular mechanisms through which EGFR inhibitors trigger autophagy have not been fully elucidated. Second, the role of autophagy may change dynamically according to extracellular environments. Third, when cancer cells undergo apoptosis followed by EGFR inhibitor treatment, they can show varying responses of activated autophagy.

Several discussion points addressing the limitations of this study should also be considered. First, this study was performed in vitro with two bladder cancer cell lines only. In order to consolidate our findings, an in vivo validation study should be conducted by using various bladder cancer cell lines. Second, to better address the clinical impact, the phenomenon of autophagy activation by EGFR inhibitors treatments should be examined in human bladder cancer specimens by using patient-derived bladder cancer xenograft models. Third, the innate drug toxicity and limited anti-cancer efficacy of autophagy inhibitors such as anti-malarial agent CQ is the critical problem for its clinical application in cancer patients. More effective and safe autophagy inhibitors should be developed in the future, thus offering a promising therapeutic strategy to overcome resistance to EGFR inhibitors and to improve the anti-cancer effects of these agents for patients with advanced stage bladder cancer.

## 4. Experimental Section

### 4.1. Cell Culture and Reagents

Human bladder cancer cell lines (T24 and J82) were purchased from the American Type Culture Collection (Rockville, MD, USA). T24 and J82 were cultured in Dulbecco’s Modified Eagle’s (DMEM) (WELGENE, Daegu, Korea), supplemented with 10% fetal bovine serum (WELGENE), 1% penicillin-streptomycin (Invitrogen, Carlsbad, CA, USA) and 1% nonessential amino acids (Invitrogen), at 37 °C with 5% CO_2_. Gefitinib (Iressa: TOCRIS, Ellisville, MN, USA) and lapatinib (Santa Cruz Biotechnology, Santa Cruz, CA, USA) were purchased as EGFR inhibitors. Bafilomycin A1 (BFA1) (AG SCIENTIFIC, San Diego, CA, USA), chloroquine (CQ) (TOCRIS) and 3-methyladenine (3-MA) (TOCRIS) were purchased as autophagy inhibitors.

### 4.2. Cell Viability Assay

EZ-Cytox (DOGEN, Seoul, Korea) was used to assess the cell viabilities under various treatment conditions. Bladder cancer cells were treated with EGFR inhibitors and/or autophagy inhibitors in 96-well culture dishes in a time- and dose-dependent manner, CCK-8 solution (10 μL) was added, and reactions were run for 2 h at room temperature (RT). The relative absorbance of each well was measured at 450 nm using a microplate reader (PerkinElmer, Waltham, MA, USA). Cell viability was presented as relative percentages, compared to the negative control. To suppress the autophagy activities by genetic manipulation, *ATG12*-siRNA (Santa Cruz Biotechnology) was transfected into human bladder cancer cells for 24–48 h. Transfected efficiency was assessed by real-time polymerase chain reaction (PCR) and Western blot analysis. For acquiring cell viability data, CCK-8 assay was performed. Scramble siRNA-transfected cells were regarded as the negative control.

### 4.3. Quantitative Real-Time PCR Analysis

Complementary DNA (cDNA) was synthesized using TOPscript^TM^ RT DryMIX (Enzynomics, Seoul, Korea) with oligo-deoxythymidine primers from total RNA of samples. To quantify the transcriptional expression of interested genes, cDNA was amplified with the primers of target genes by using the SDS 7500 FAST Real-time PCR system (Applied Biosystems, Foster City, CA, USA) with EvaGreen qPCR Mastermix (ABM Inc., Richmond, BC, Canada). Relative expression of target genes was calculated using the 2^−ΔΔ*C*t^ method. The glyceraldehyde-3-phosphate dehydrogenase gene (GAPDH) and 18S ribosomal RNA were used as endogenous reference genes for normalization.

### 4.4. Western Blot Analysis

Cells were lysed in RIPA buffer (150 mM NaCl, 50 mM Tris-HCl (pH 7.2), 0.5% NP-40, 1% Triton X-100, 1% sodium deoxycholate) containing a protease inhibitor cocktail (Sigma-Aldrich Corporation, St. Louis, MO, USA). Cell lysates were separated on sodium dodecyl sulfate (SDS)-polyacrylamide gels and transferred to an Immobilon-P membrane (Millipore, Darmstadt, Germany). Membranes were blocked with 5% skim milk and 0.1% Tween-20 for 1 h and incubated overnight at 4 °C with the indicated primary antibodies. Membranes were incubated with a horseradish peroxidase–conjugated secondary antibody (1:5000) for 1 h and developed using the ECL-Plus Kit (ThermoFisher Scientific, Waltham, MA, USA). Primary antibodies used are listed on Table 1.

### 4.5. Flow Cytometry Analysis of Apoptosis

The annexin-V-FITC/PI double staining assay was performed to examine the apoptotic cell death under various treatment conditions. Samples were washed with phosphate-buffered saline (PBS) three times and stained using the FITC Annexin V Apoptosis Detection Kit (BD Biosciences, San Jose, CA, USA) for 15 min at RT, according to the manufacturer’s protocol. The number of apoptotic cells was analyzed by flow cytometry (BD FACSCalibur™ cytometer; BD Biosciences).

### 4.6. Colony Formation Assay

Cells (1.5 × 10^3^/well) were seeded onto 6-well plates and cultured for 14 days to form visible colonies. After colonies were fixed with the 10% neutral buffered formalin solution (Sigma-Aldrich Corporation), they were stained with a 0.01% crystal violet solution. After rinsing 3 times, visible colonies comprised of more than 50 individual cells were counted using a SZX7 stereo microscope (Olympus, Tokyo, Japan).

### 4.7. Generation of LC3-GFP-Expressing T24 Cells and Monitoring of Autophagy Activation

Mammalian expression plasmids containing the human LC3B protein (pSELECT-GFP-LC3) were purchased from InvivoGen (San Diego, CA, USA). The DNA was transfected into T24 cells using Lipofectamine 2000 (ThermoFisher Scientific). The cells containing LC3B plasmid were selected with Zeocin (100 μg/mL) for 3 weeks, and the expression was confirmed with GFP fluorescence under fluorescent microscopy after LC3 induction.

To visualize the autophagy activation, LC3-GFP expression T24 cells were plated and cultured on 4-well Nunc^™^ Lab-Tek^®^ Chamber Slides (ThermoFisher Scientific) for 2 days. Initial images of cells were obtained at the basal layer, before being treated for 24 h with EGFR inhibitors and acquiring live pictures using a confocal laser scanning microscope (Leica, Wetzlar, Germany). To identify the location of nuclei, samples were mounted with VECTASHIELD Mounting Medium containing 4′,6-diaminidine-2-phenylindole (1:1000) (Vector Laboratories, Burlingame, CA, USA). LC3-GFP-expressing T24 cells cultured in serum starvation conditions for 24 h were used as a positive control of autophagy activation.

### 4.8. Statistical Analysis

All experiments were conducted in triplicate to assess statistical significance. The values in the bar graph are shown as the mean ± standard error of the mean (SEM). Significance differences between two groups were determined using a Student’s *t*-test. *P*-values less than 0.05 were considered statistically significant. GraphPad Prism software (GraphPad Software Inc., San Diego, CA, USA) and the SPSS software Version 22.0 (IBM, Armonk, New York, USA) were used to analyze all experimental data.

## 5. Conclusions

In summary, EGFR inhibitors significantly induced autophagy activities in human bladder cancer cells, and concomitant suppression of autophagy activities enhanced the anti-cancer effects of EGFR inhibitors, such as lapatinib and gefitinib. Our work suggests a potentially promising therapeutic approach to treat patients with advanced stage bladder cancer.

## Figures and Tables

**Table 1:** Primary antibodies used in the study.

Antibody	Host	Dilution Factor	Industry
LC3 (microtubule-associated protein light chain 3)	Rabbit	1:4000	Novus Biologicals
β-Actin	Rabbit	1:10,000	Sigma-Aldrich Corporation
ATG5 (Autophagy protein 5)	Rabbit	1:1000	Cell Signaling Technology
p-EGFR (phosphorylated-epidermal growth factor receptor)	Rabbit	1:1000	Santa Cruz Biotechnology
EGFR (epidermal growth factor receptor)	Rabbit	1:2000	Santa Cruz Biotechnology

## Supplementary Materials

Supplementary materials can be found at www.mdpi.com/1422-0067/18/2/321/s1.
