# Supplementary material for: Concurrent Autophagy Inhibition Overcomes the Resistance of Epidermal Growth Factor Receptor Tyrosine Kinase Inhibitors in Human Bladder Cancer Cells
Source: Int J Mol Sci. 2017 Feb 4;18(2):321. doi: 10.3390/ijms18020321 (PMC5343857; doi:10.3390/ijms18020321)
Supplement: Supplementary file 1 [file ijms-18-00321-s001.pdf]

Supplementary Materials: Concurrent Autophagy Inhibition Overcomes the Resistance of Epidermal Growth Factor Receptor Tyrosine Kinase Inhibitors in Human Bladder Cancer Cells

Minyong Kang, Kyoung-Hwa Lee, Hye Sun Lee, Chang Wook Jeong, Cheol Kwak, Hyeon Hoe Kim and Ja Hyeon Ku

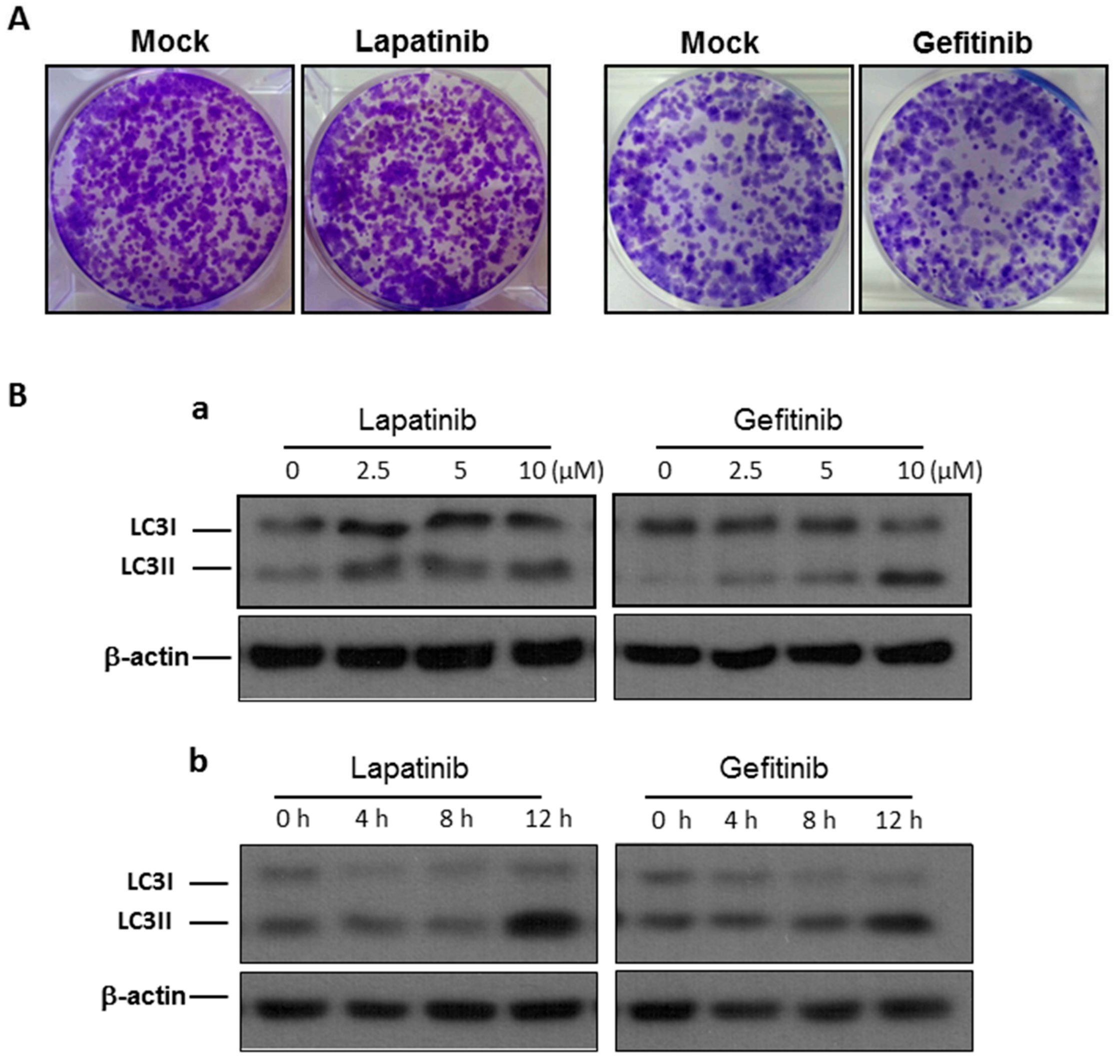

Figure S1. Non-significant anti-cancer effects of epithelial growth factor receptor (EGFR) inhibitors on J82 human bladder cancer cells and autophagy activation. (**A**) clonogenic assay evaluating the anti-cancer effects of lapatinib and gefitinib (5 μM) for 10 to 14 days on J82 human bladder cancer cells. (**B**) Western blot analysis of autophagy activation by determining the expression of LC3I/II under (**a**) different dosages (0, 2.5, 5 and 10μM) and (**b**) different treatment times (0, 4, 8 and 12 h).

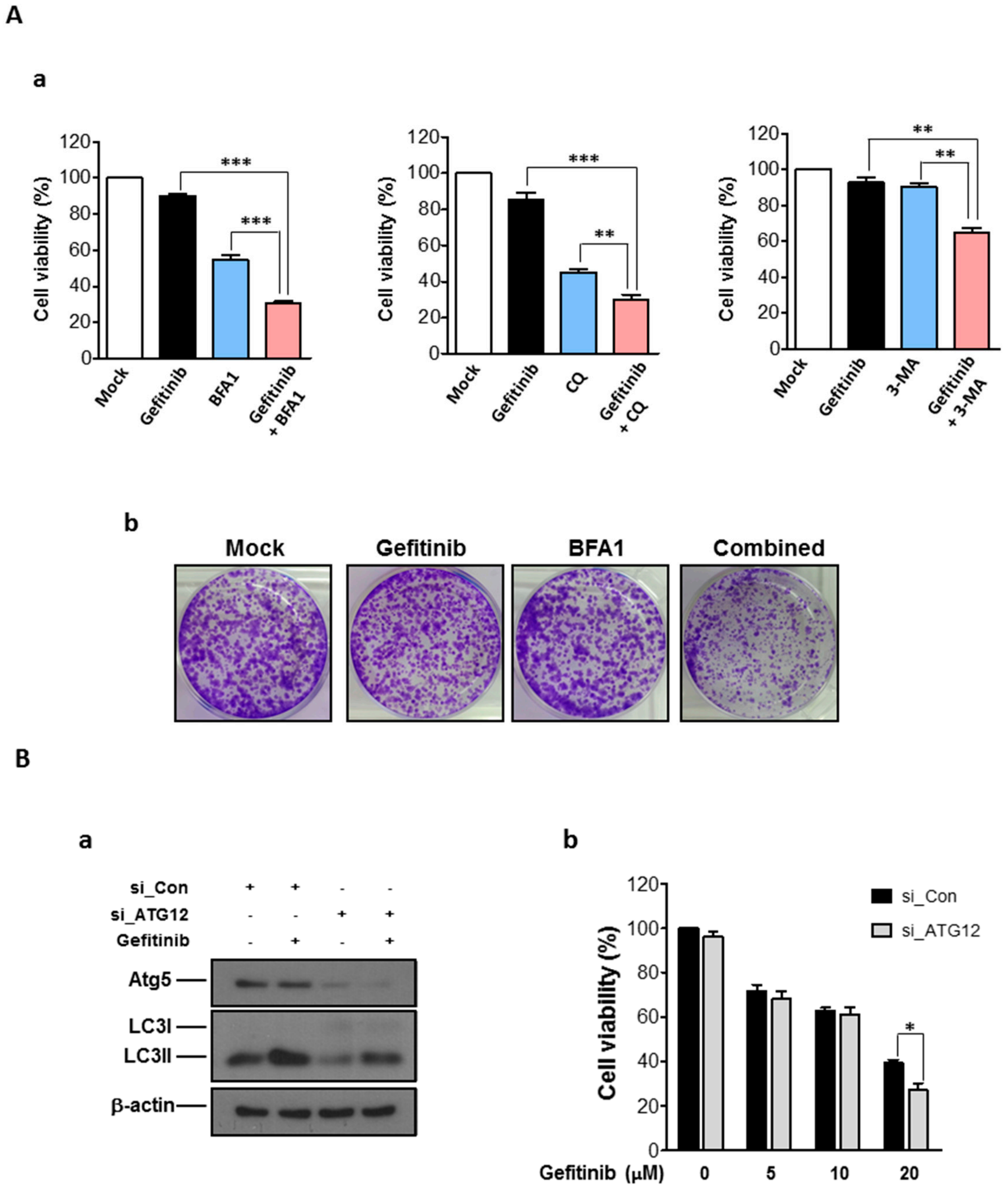

Figure 2. Synergistic anti-cancer effects of EGFR inhibitors and autophagy inhibition on J82 human bladder cancer cells. (**A**) Evaluation of anti-cancer effects of gefitinib (5μM) in J82 human bladder cancer cells by performing cell viability analysis under different treatment combinations (control, gefitinib, autophagy inhibitors and both agents). The autophagy inhibitors used in this study were 5 nM of bafilomycin A1 (BFA1), 10 μM of chloroquine (CQ) and 10 mM of 3-methyladenine (3-MA). Cell viability is shown as the mean percentage (%) of the control ± SEM ($n = 3$, ** $p < 0.01$, *** $p < 0.001$). (**B**) (**a**) Western blot analysis to determine the knock-down efficiency and autophagy suppression by transfection of ATG12-siRNA (si_ATG12) (50 nM) for 48 h in J82 cells, combined with 5 μM of gefitinib treatment. Scramble siRNA (si_Con) (50 nM) was used as a negative control of si_ATG12. (**b**) Under the same treatment conditions of gefitinib (5 μM) and 50 nM of si_ATG12 for 48 h, the cell viability assay was performed and data are represented by the mean percentage of the control ± SEM ($n = 3$, * $p < 0.05$).
